# Living Alone among Older Persons in Uganda: Prevalence and Associated Factors

**DOI:** 10.1007/s12126-017-9305-7

**Published:** 2017-11-18

**Authors:** Stephen Ojiambo Wandera, Isaac Ddumba, Joshua Odunayo Akinyemi, Sunday A. Adedini, Clifford Odimegwu

**Affiliations:** 1 Department of Population Studies, School of Statistics and Planning, College of Business and Management Sciences, Makerere University, Kampala, Uganda; 2 Demography and Population Studies Programme, Schools of Social Sciences and Public Health, University of the Witwatersrand, Johannesburg, South Africa; 3 Department of Health, Mukono District Local Government, Mukono, Uganda; 4 Department of Epidemiology and Medical Statistics, Faculty of Public Health, College of Medicine, University of Ibadan, Ibadan, Nigeria; 5 Department of Demography and Social Statistics, Faculty of Social Sciences, Obafemi Awolowo University, Ile-Ife, Nigeria

**Keywords:** Living arrangements, Elderly, Uganda, Sub-Saharan Africa

## Abstract

This study aimed at investigating the prevalence and factors associated with living alone among older persons in Uganda. A secondary analysis of the 2010 Uganda National Household Survey (UNHS) data was conducted. A complementary log-log regression model was used to estimate the association between living alone and demographic, socio-economic and health factors. Nearly one out of ten (9%) older persons lived alone in Uganda. Living alone was associated with being divorced / separated (OR 18.5, 95% CI: 10.3–33.3), being widowed (OR 8.8, 95% CI: 5.1–15.2), advanced age (OR 2.1, 95% CI: 1.4–3.2), residence in western region (OR 0.6, 95% CI: 0.3–0.93), poor wealth status (OR 0.3, 95% CI: 0.2–06), receiving remittances (OR 1.6, 95% CI: 1.1–2.3) and being disabled (OR 1.6, 95% CI: 1.2–2.1). Living alone among older persons did not vary by gender.

## Introduction

Population ageing has become a global concern in the past two decades (Beard et al. 2015). Improvement in the health care systems, decreased fertility rates and reduction in child mortality have contributed to the phenomenon (Bloom 2011; Cai 2010). There is a remarkable variation across continents, regions and countries with Western countries contributing to the majority of the older population (Guzman et al. 2012).

Worldwide, the proportion of older persons (aged 60 years and above) stands at 11% and it’s anticipated to double by 2050 (UNDESA 2013). In sub-Saharan Africa, older persons comprise 5% of the population (UNFPA 2012). In Uganda, the current population of older persons is estimated at 1.6 million (5% of the population) and it is expected to increase to 5.5 million in 2050 (UBOS and ICF International 2012).

Living arrangements is a critical issue in the discourse on population ageing. Living arrangements depict familial and non-familial relationships with whom the older persons share / reside in the same household (Victor 2005). Living arrangements provide immediate and adjacent social care and support to frail older persons (Moen and Wethington 1992). Living alone is where an older person lives alone in a household.

In the past two centuries, patterns of living arrangements among older persons in the western world have drastically changed (Bongaarts and Zimmer 2002). Trends of older persons living alone have steadily increased (Hays and George 2002; Kramarow 1995; Zsembik 1993). Studies have documented that improvement in the housing structure (Schafer 1999), need for privacy in later years (Portacolone and Halpern 2014), departure of adult children due to marriages, widowhood and divorces are associated with living alone in later years (Gibler et al. 1998; Kim and Rhee 1997; Wolf 1995). For example in Japan, 15% of older persons were living alone in 2005 (NIA 2017). In Norway, most of the older persons lived in single households (Tomstad et al. 2012).

The proportion of older persons living alone varies from continent to another. A comparative study of living arrangements among older persons indicated that the percentage of those living alone was higher in Africa (2%) than in Asia (1%) and Latin America (1.4%). Among those age 65+, the percentage living alone was higher in Africa (9.7%) compared to Asia (7.3%) and Latin America (8.4%). Generally, the prevalence of older women living alone was higher than that of older men in the three continents (Bongaarts and Zimmer 2002).

Living alone poses a high risk of poor health conditions and outcomes among older persons (Cornwell and Waite 2009; Haslbeck et al. 2012; Magaziner et al. 1988). Most studies have documented that living alone leads to poor psychological wellbeing (Dykstra 2009; Lim and Kua 2011; Millan-Calenti et al. 2013), depression (Mui 1999; Russell and Taylor 2009), higher mortality (Iecovich et al. 2011) and loneliness (Nzabona et al. 2015) among older persons.

In addition, living alone threatens the financial and social wellbeing of older persons in later years (Sereny 2011; Wandera et al. 2015b; Zimmer 2005). For instance, in Uganda, the social security scheme for older persons is partially developed, selected districts receive unconditional cash transfers (MoGLSD 2009, 2011). Currently, Uganda is implementing the social assistance grants for empowerment (SAGE grant) where some older persons (age 65 and older), receive an equivalent of $8 per month. However, this scheme is only operational in about 14 out of 112 districts in Uganda (MoGLSD 2009, 2011). Therefore, families and kinships are primarily responsible for supporting older persons in their later years (Lloyd-Sherlock and Agrawal 2014). Due to the relevance of the family in social support, it is imperative to investigate factors associated with living alone among older people.

## Factors Associated with Living Alone

Generally, factors associated with living alone among older persons include being very old, being female, urban residence, lower socio-economic groups, not having a partner, poor health, or living without a child nearby (Bongaarts and Zimmer 2002; Hosegood and Timæus 2005; Isherwood et al. 2012; Lawton et al. 1984; Nahemow 1979; Nzabona et al. 2015; Peek et al. 1997). Higher socio-economic status was associated with living alone in China (Sereny 2011).

In addition, the HIV/AIDS pandemic has virtually left most of the older persons to reside alone or with grandchildren due to loss of their adult children (Seeley et al. 2010; Seeley et al. 2009; Ssengonzi 2009). In addition, being childless (Schroder-Butterfill and Kreager 2005), having adult daughters leaving the household due to marriage and participation in labor force (Qin et al. 2008), formation of nucleated family, modernization and erosion of traditional social cohesion (Agree et al. 2005) have also been associated with living alone among older persons in later years.

Studies on living arrangements among older persons have majorly focused on East and south East Asia (Chaudhuri and Roy 2009; Chen et al. 2016). Some studies have focused on Latin America (de Vos 2000) and very few on Africa (Bongaarts and Zimmer 2002). The study on Africa included Uganda and used the Uganda Demographic and Health Survey (UDHS) data of 1990–1998.

In Uganda, few studies have addressed loneliness (Nahemow 1979; Nzabona et al. 2015), social isolation (Nahemow 1979; Richards 1966) and living arrangements of older persons. Some of these studies used small samples of older people among the Baganda tribe (Bennett and Mugalula-Mukiibi 1967; Nahemow 1979; Richards 1966). One of the Ugandan studies on living alone was among the Ganda community but had limited focus on older people (Bennett and Mugalula-Mukiibi 1967). The recent study on loneliness covered a sample of 605 older people, and did not conduct a gender-disaggregated analysis (Nzabona et al. 2015). Therefore, this paper aimed at investigating the prevalence and factors associated with living alone among older persons in using a nationally representative sample (*n* = 2382) of older persons in Uganda.

## Methods

### Data Source

We used the 2010 Uganda National Household Survey (UNHS) data, with permission from Uganda Bureau of Statistics (UBOS). The UNHS employed a two-stage stratified sampling. At the first stage, 712 enumeration areas were drawn using a probability proportionate to size. The second phase, all households were selected using systematic sampling. About 6800 households were interviewed in the survey. Detailed sampling procedures are reported elsewhere (UBOS 2010).

In this paper, older persons were defined as those aged 50 years and above. This definition is recommended by the World Health Organization (WHO) for African countries (WHO 2015). Several “Studies on Adult health and Ageing” (SAGE) and INDEPTH network have used age 50+ to define older persons (Gómez-Olivé et al. 2010; Gómez-Olivé et al. 2013; Mwanyangala et al. 2010; Ng et al. 2010a; Ng et al. 2010b; Nyirenda et al. 2012) .

### Outcome Variable

Studies on living arrangements of older persons use the household as a unit of analysis and the household composition (household listing) variable from census and survey data including the Demographic and Health Survey (DHS) (Bongaarts and Zimmer 2002; Cheng and Siankam 2009; Yount and Khadr 2008). Household composition includes information about household members living in the household. The most commonly used coding categories for living arrangements among older people include living alone, living with a partner and living with others. For example, co-residence with adult children (Nguyen and Shibusawa 2013; Ugargol et al. 2016).

In this paper, living alone was the outcome variable. In the 2010 UNHS, respondents were asked to list all the household members in the last 12 months (including the guests) in the household roster. This was used to derive the outcome variable dichotomized as (0 – living with 1 or more people and 1 – living alone). In Uganda, a household is defined as people who live (sleep) and eat together (UBOS 2010).

### Explanatory Variables

Demographic factors included; gender (male or female), age group (50–59, 60–69, 70–79, 80+) and region (1-Central, 2-Eastern, 3-Nothern and 4-Western). Others included; place of residence (rural or urban), and marital status (1-Married, 2-separated or divorced or never married, and 3-widowed).

In addition, respondents were asked to specify the relationship of each household member to the household head. Different relationships included: head, spouse, son/daughter, grandchild, step child, parent of head or spouse, sister/brother of head or spouse, nephew/niece, other relatives, servant, non-relative, others) (UBOS 2010). In this paper, all relationships excluding household head and being a spouse were coded as relatives.

Socio-economic factors included; level of education, household poverty status, and household major source of earnings. The level of education was coded as: no education, primary, secondary and tertiary education. Household poverty was generated from household expenditures and recoded (1-poor, if the household spent less than a $1 a day and 0-not poor, if a household spent greater than a $1 a day). The households’ major sources of income were recoded as farming, wages, and remittances. These have been described elsewhere (Wandera et al. 2015a; Wandera et al. 2015b; Wandera et al. 2014).

Health related information was collected on illness in the last 30 days prior the survey. Ill health was binary variable (0-not sick, 1-sick). Disability was measured by asking six questions including difficulties in seeing, walking, hearing, communicating, concentrating or remembering and self-care. Disability status was generated from these six indicators using the UN Washington Group meeting on disability statistics. An older person was coded as disabled when they reported a lot of difficulty or could not function on any one of the six indicators. In addition, reporting some difficulty on at least two indicators was coded as being disabled.

In addition, self-reported NCDs were subjectively asked among older people. In the UNHS survey, respondents were asked to report whether they had any one of the three NCDs: hypertension, diabetes, or heart disease. The question allowed multiple responses to these three health conditions. These responses were coded as binary (0 = did not report any NCDs and 1 = reported at least one of the three NCDs. Thus, self-reported NCDs meant the reporting of diabetes, heart disease and hypertension, recoded as binary variable.

### Statistical Analysis

We used the Pearson chi-square tests to identify initial bivariate associations and multivariable complementary log-log regression to estimate factors associated with living alone. The complementary log-regression model is recommended for rare outcomes (9% of older persons living alone) or when data is symmetrical and the outcome is binary (Long 1997; Penman and Johnson 2009). All the analyses were stratified by gender. We included variables that were significantly (*p* = 0.05) associated with living alone among all older persons. We used Stata version 13 to analyze the data. The survey was weighed to account for the complex survey design.

## Results

### Descriptive Characteristics of Older Persons in Uganda

Table 1 presents the descriptive characteristics of older persons in Uganda. Majority were women (57%) and of age 50–59 years (45%). Eastern region had a slightly higher (31%) distribution than other regions and over 90% of the older persons resided in the rural areas. More than half (59%) of older people were married.

**Table 1:** Percent distribution of older persons in Uganda stratified by gender

	Men (n = 1136)		Women (n = 1246)		All (n = 2382)	
	Number (n)	Percent (%)	Number (n)	Percent (%)	Number (n)	Percent (%)
Gender						
Male					1136	43.2
Female					1246	56.7
Age group						
50–59	524	46.1	542	43.5	1066	44.7
60–69	313	27.6	356	28.6	670	28.1
70–79	206	18.1	228	18.3	433	18.2
80+	94	8.2	120	9.6	213	9.0
Region						
Central	278	24.5	311	24.9	589	24.7
Eastern	371	32.6	358	28.7	728	30.6
Northern	216	19.1	253	20.3	470	19.7
Western	271	23.8	324	26.0	595	25.0
Place of residence						
Rural	1032	90.8	1131	90.7	2162	90.8
Urban	104	9.2	115	9.3	220	9.2
Marital status						
Married	919	80.9	477	38.3	1396	58.6
Divorced / separated	84	7.4	158	12.7	242	10.2
Widowed	133	11.7	611	49.0	744	31.2
Education level						
None	716	63.0	904	72.6	1620	68.0
Primary	304	26.8	284	22.8	588	24.7
Secondary +	116	10.2	58	4.7	174	7.3
Poverty status						
Non-poor	883	77.7	955	76.7	1838	77.2
Poor	253	22.3	291	23.3	544	22.8
Relationship to household head						
head	995	87.6	669	53.7	1664	69.9
spouse	72	6.3	387	31.0	458	19.2
relative	69	6.1	191	15.3	260	10.9
Household major source of earnings						
Farming	720	63.4	731	58.7	1451	60.9
Wages	330	29.0	307	24.7	637	26.7
Remittances	86	7.6	208	16.7	294	12.3
Was ill or injured during past 30 days						
No	495	43.6	409	32.8	904	38.0
Yes	641	56.4	837	67.2	1478	62.0
Disabled						
No	823	72.4	777	62.4	1600	67.2
Yes	314	27.6	469	37.6	782	32.8
Reported a non-communicable disease						
No	949	83.6	878	70.4	1827	76.7
Yes	187	16.4	368	29.6	555	23.3
Living alone						
No	1022	90.0	1145	91.9	2167	91.0
Yes	114	10.0	101	8.1	215	9.0

Nearly three quarters (70%) had no formal education and more than three quarters (77%) spent more than one dollar a day. Six in ten (61%) of the older persons depended on farming as the major source of household earnings.

About 6 in 10 older persons reported illness or injury in the past 30 days and a third (33%) were disabled. Similarly, near a quarter (23%) reported at least one of the non-communicable diseases including diabetes, heart disease and hypertension.

### Association between Living Alone and Selected Demographic and Socio-Economic Factors

Table 2 presents the association between living alone and demographic and socio-economic factors. Among these variables, gender, place of residence, educational level, and self-reported NCDs were not associated with living alone among older people in Uganda. However, age, region of residence, marital status, poverty status, major source of household earnings, ill health and disability status, were associated with living alone among older people in Uganda.

**Table 2:** Percentage of living alone among older persons in Uganda by socio-demographic factors, stratified by gender

	Men			Women			All		
	(n)	% Living Alone	p-value	(n)	% Living Alone	p-value	(n)	% Living Alone	p-value
Gender									0.12
Male							1136	10.0	
Female							1246	8.1	
Age group			p < 0.01			p < 0.01			p < 0.01
50–59	524	6.2		542	4.0		1066	5.0	
60–69	313	10.9		356	8.9		670	9.8	
70–79	206	16.4		228	15.2		433	15.8	
80+	94	14.9		120	11.0		213	12.7	
Region			p < 0.01			0.03			p < 0.01
Central	278	16.1		311	10.4		589	13.1	
Eastern	371	10.1		358	8.8		728	9.4	
Northern	216	7.9		253	9.4		470	8.7	
Western	271	5.4		324	4.1		595	4.7	
Place of residence			0.31			0.46			0.21
Rural	1032	9.7		1131	7.9		2162	8.7	
Urban	104	13.9		115	9.8		220	11.8	
Marital status			p < 0.01			p < 0.01			p < 0.01
Married	919	1.9		477	0.7		1396	1.5	
Divorced / separated	84	53.3		158	19.1		242	30.9	
Widowed	133	38.7		611	11.0		744	16.0	
Education level			0.99			0.37			0.75
None	716	10.1		904	7.9		1620	8.9	
Primary	304	9.9		284	9.5		588	9.7	
Secondary +	116	10.0		58	4.2		174	8.1	
Total	1136	10.0		1246	8.1		2382	9.0	
Poverty status			p < 0.01			p < 0.01			p < 0.01
Non-poor	883	11.5		955	9.8		1838	10.6	
Poor	253	4.9		291	2.5		544	3.6	
Relationship to household head			p < 0.01			*p* < 0.01			p < 0.01
Head	995	11.5		669	15.1		1664	12.9	
Spouse	72	0.0		387	0.0		458	0.0	
Relative	69	0.0		191	0.0		260	0.0	
Household major source of earnings			p < 0.01			p < 0.01			p < 0.01
Farming	720	6.7		731	6.4		1451	6.5	
Wages	330	13.4		307	6.1		637	9.9	
Remittances	86	25		208	17.1		294	19.4	
Was ill or injured during past 30 days			0.02			0.01			p < 0.01
No	495	7.6		409	4.9		904	6.4	
Yes	641	11.9		837	9.6		1478	10.6	
Disabled			p < 0.01			p < 0.01			p < 0.01
No	823	6.9		777	5.2		1600	6.1	
Yes	314	18.2		469	12.9		782	15.0	
Reported at least one NCD			0.35			0.47			0.81
No	949	10.4		878	7.7		1827	9.1	
Yes	187	8.2		368	9.0		555	8.7	
Total	1136	10.0		1246	8.1		2382	9.0	

The prevalence of living alone was higher among older people with advanced age (70–79+), residing in central region, and those who were divorced or separated. In addition, living alone was also high among older persons who were disabled, depended on remittances, and reported ill health in the last 30 days.

### Multivariable Results

Table 3 shows the results of multivariable complementary log-log regression of factors associated with living alone among older persons in Uganda.

**Table 3:** Results of the complementary log-log regression of living alone on selected socio-demographic factors among older persons in Uganda

	Men		Women		All	
Variables	Odds Ratios	95% CI	Odds Ratios	95% CI	Odds Ratios	95% CI
Age group (rc = 50–59)						
60–69	1.36	[0.79–2.34]	1.64	[0.90–3.00]	1.53^*^	[1.04-2.23]
70–79	1.27	[0.67–2.43]	2.44^**^	[1.27-4.69]	2.10^***^	[1.39-3.17]
80+	1.04	[0.49–2.18]	1.46	[0.70–3.06]	1.45	[0.88–2.38]
Region (rc = Central)						
Eastern	0.77	[0.45–1.30]	1.21	[0.69–2.11]	1.01	[0.68–1.50]
Northern	0.61	[0.32–1.15]	1.87^*^	[1.03-3.37]	1.13	[0.73–1.76]
Western	0.42^**^	[0.22-0.80]	0.69	[0.33–1.45]	0.55^*^	[0.33-0.92]
Marital status (rc = Married)						
Married	1	[1–1]	1	[1–1]	1	[1–1]
Divorced or separated	28.9^***^	[14.7–57.0]	24.0^***^	[7.48-77.0]	18.5^***^	[10.3-33.3]
Widowed	24.8^***^	[13.6-45.0]	11.2^***^	[3.52-35.4]	8.80^***^	[5.10-15.2]
Poor (rc = No)	0.43^*^	[0.20–0.93]	0.22^***^	[0.095-0.50]	0.32^***^	[0.18-0.57]
Major household source of earnings (rc = Farming)						
Wages	1.61^*^	[1.04-2.51]	0.91	[0.54–1.54]	1.32	[0.95–1.85]
Remittances	2.43^**^	[1.31-4.51]	1.77^*^	[1.07-2.92]	1.59^*^	[1.11-2.26]
Was ill or injured during past 30 days (rc = No)	1.04	[0.67–1.61]	1.32	[0.74–2.35]	1.09	[0.78–1.52]
Disabled (rc = No)	1.32	[0.86–2.04]	1.75^*^	[1.11-2.75]	1.56^**^	[1.15-2.10]
Observations	1241		1387		2628	
Link test						
_hat (p)	0.003		0.047		0.001	
_hatsq (p)	0.074		0.269		0.316	

CI = Confidence Intervals

^*^ *p* < 0.05,

^**^ *p* < 0.01

^***^ *p* < 0.001

The odds of living alone increased with age (70–79) among older women and not for older men. There were no differences in living alone among all older persons aged 50–59 and 80+ years. Advancement in age was not associated with living alone among older men. However, older age was associated with living alone among women.

Older persons from western compared to central region were less likely to live alone. This was the same pattern for older men. For older women, those from northern region were more likely to live alone compared to those from central region.

Separated, divorced, and widowed older persons had increased odds of living alone. The same pattern was observed for both older men and women.

Older persons who were poor had reduced odds of living alone. The same pattern was observed for men, women and all older persons. Older people who depended on remittances were more likely to be living alone than those who depended on farming. Older men who earned wages were more likely to live alone compared to those who depended on farming.

In addition, women and all older people who reported a disability had increased odds of living alone. However, disability status was not associated with living alone among older men only.

## Discussion

The prevalence of living alone among older persons in Uganda was 9%. This is about the same level as that of 23 selected African countries including Kenya, Tanzania, Rwanda and Uganda among others (Bongaarts and Zimmer 2002). Although in the developing countries, the phenomena of living alone is uncommon, the structure of living arrangement is starting to shift and this is expected to change in the next two decades (Bloom 2011; Eastwood and Lipton 2011). This is higher than the prevalence of living alone in other developing countries like India (Ugargol et al. 2016) and lower than that (over 50%) reported in China (Nguyen and Shibusawa 2013).

The factors which were strongly associated with living alone were marital status and being poor (negative association). Others included age, region of residence, source of household earnings, being disabled but not ill health in the last 30 days.

Marital status had the strongest association with living alone among older persons. Separated, divorced or widowed older persons had increased odds of living alone in Uganda. This confirms the findings in a Ugandan study (Nzabona et al. 2015) and elsewhere (Lawton et al. 1984; Mba 2002, 2007). The bivariate results (Table 2) indicated that more divorced / separated men (53%) than women (19%) were living alone. Older women usually care for orphans as result of HIV (Seeley et al. 2010; Seeley et al. 2009).

Low socio-economic status or poverty decreased the odds of living alone. Poor older persons (who spent less than a dollar per day) were less likely to live alone. In other words, those in better socio-economic status were more likely to live alone. This resonates with the study in China where older persons who were in a better socio-economic status preferred to live independently (Sereny 2011). In addition, those who depended on remittances were more likely to be living alone. Older persons who receive regular remittances are economically empowered and therefore, are more likely to live alone in (Hosegood and Timæus 2005). Generally, the need for social protection among older persons influences co-residence with adult children in later life (Lloyd-Sherlock 2001, 2002; Lloyd-Sherlock and Agrawal 2014). In Uganda, poverty is common among older persons and their vulnerability creates inequitable access to services (Golaz and Rutaremwa 2011). Since over 70% of older person had informal education and most of them depended on farming, their income is quite low hence increasing their vulnerability to poverty. A study in Uganda indicated that absence of pension benefits was associated with loneliness among older persons (Nzabona et al. 2015).

Advancement in age (70–79) increased the risk of living alone in Uganda. Increment in age reduces physical abilities and health status, which necessitate the need for assistance through co-residence (Liang et al. 2005; Wandera et al. 2015a). Wandera et al. highlighted the risk of disability; non-communicable diseases with poor health outcomes were more common among the oldest old (Wandera et al. 2015b; Wandera et al. 2014). Living alone could be associated with stigma or HIV/AIDS epidemic which leave older persons without relatives to co-reside with (Seeley et al. 2009). This was a bigger issue for older women than older men. Older women were at a higher risk of living alone because of advanced age. Women have a higher life expectancy and are more likely to be widowed with advancement in age compared to older men.

Region of residence was associated with living alone among older persons in Uganda. Older persons in western region were less likely to live alone compared to those in central Uganda. Similarly, older men were less likely to live alone in western region compared to western region. The possible explanation is the communal culture in western Uganda since some are pastoralists for example the Banyankole. On the other hand, central Uganda includes Kampala, which is a capital city and other neighboring towns. Older persons in urban areas face a higher risk of being isolated and living alone (Bennett and Mugalula-Mukiibi 1967; Nzabona et al. 2015). Another explanation for older people in central Uganda living alone more than other regions, is the cultural issue. In central Uganda, adult children prefer to establish homes away from their parents in order to be independent (Nahemow 1979; Richards 1966). Among older women only, those in northern region were more likely to live alone compared to those in central region. This resonates with the effect of the 20 years’ civil war in the region, which left many men dead and increase widowhood among them (Annan et al. 2011).

Disabled older persons were more likely to live alone compared to those who were not. Disability and old age are highly stigmatizing experiences associated with a high degree of vulnerability (Golaz and Rutaremwa 2011) and call for assistance in form of co-residence from kins (Stinner et al. 1990). It is common to find older persons who are isolated and living alone and assumed to be “witches” by the community around (Nzabona et al. 2015). A study in India also found that disabled older persons were more likely to live alone as result of social isolation and deprivation from the family (Ugargol et al. 2016). Similar findings were reported in china (Nguyen and Shibusawa 2013). In addition, the strains and burden of caring for disabled older persons are high on the care givers as postulated in the “privacy model” (Stinner et al. 1990; Ugargol et al. 2016) and therefore, family members could avoid them for such a reason.

Gender plays a crucial role in older persons’ living arrangements. Older women are reported to live alone more than older men do elsewhere (Bongaarts and Zimmer 2002; Chaudhuri and Roy 2009; Peek et al. 1997; Yount and Khadr 2008). However, for this study, there were no significant differences in the prevalence of living alone between older men (10% vs 8%) and women (Table 2). This contradicts with findings in other studies that report older men living alone more than older women (Isherwood et al. 2012). A study in the US reported that older women lived alone more than older men since the former had more connections with adult children than the later (Gaymu and Springer 2010). The possible explanation is that older women are more likely to live with grandchildren as care givers in the context of HIV and AIDS (Seeley et al. 2010; Seeley et al. 2009; UNDESA 2005). On the other hand, older men are more likely to remarry after widowhood, separation and divorce (Bongaarts and Zimmer 2002; Silverstein et al. 2006; Utz et al. 2002). In addition, it is a possibility that cultural factors or social desirability could have influenced women’s under reporting of living alone. In Uganda, women who live alone are considered as “social misfits” in society.

Ill health or being sick in the last 30 days was not associated with living alone. Some study in Uganda found that living alone was associated with poor health status (Nahemow 1979). The explanation was that people who are live alone / isolated or lonely tend to view their health status as bad (Cornwell and Waite 2009; Nahemow 1979). Thus poor self-rated health might be connected to social isolation. In the UNHS data, respondents were not asked to rate their health status. In future surveys, adding such questions would be relevant. In addition, older persons who fall ill can be taken for healthcare by their adult children (Zimmer 2005), who might be living in urban areas, where care is perceived to be better. Critical variables like the influence of Human Immuno-Deficiency Virus (HIV) pandemic were not captured in the UNHS and therefore, it was not possible to analyze them.

### Strength and Limitations

The strength of this paper is that it contributes to knowledge about the prevalence, demographic, health and socio-economic factors associated with living alone among older persons using a nationally representative sample (2010 UNHS data) in Uganda. Household based surveys including the Demographic and Health Surveys (DHS) have been used in the study of older persons (Bongaarts and Zimmer 2002). Notwithstanding the strength of the paper, several limitations merit discussion.

First, data from household surveys (including UNHS) has constraints in studying the living situations of older people. In the UNHS, data on the number of living children of older people who live outside the household was not collected. Thus, the proximity of children who don’t co-reside with older people is not known. The data cannot tell us who else was living in the proximity of the older persons. Also, exchanges between children and older people is not known (Bongaarts and Zimmer 2002). However, in absence of national surveys on older people, such data are still useful in gleaning out living arrangements of older people.

Second, we used cross sectional data, which do not allow investigating patterns of living arrangements over time. They only provide statistics of living arrangements at a point time without critically analyzing how living alone vary with other variables over the years. Hence, investigation of causal factors contributing to living alone among older person is not feasible. However, the data provides the snapshot about the prevalence and gravity of the problem among the older persons in Uganda.

Third, the UNHS did not clearly depict the marital status of the adult children; this has an implication towards the co-residence with older persons. As per cultural consideration in Uganda, married couples leave their parents to co-reside with other partners. Hence, it was hard to ascertain whether the older persons were childless or had adult children who left the household due to other reasons.

### Conclusions

In Uganda, living alone was associated with advanced age, living in central region, widowhood, marital separation and divorce, being in a better socio-economic status, depending on remittances and being disabled. Surprisingly, there was no significant difference in living alone among older persons by gender.

Due to the vulnerability associated with living alone, there is a need to formulate policies and design programs that create formal community care centers for older persons who live alone and have no extended family support networks in Uganda. In most developing countries, governments and policy makers tend to value familial systems as source of protection for older persons in later years. However, the change in traditional social cohesion across generations points to a need to strengthen social support systems in later years.

There is a need to enact policies that address poverty and inequality among older persons in Uganda. Although, there is an on-going social assistance grants for empowerment (SAGE), which provides an equivalent of ten dollars to older persons per month, the target beneficiaries should be those who are living alone. The SAGE program targets selected 14 out of 112 districts in Uganda.
